# Association between hs-CRP Levels and the Outcomes of Patients with Small-Artery Occlusion

**DOI:** 10.3389/fnagi.2016.00191

**Published:** 2016-08-09

**Authors:** Ruiying Qiu, Yuan Gao, Dongzhe Hou, Yajing Wang, Changshen Yu, Wanjun Wang, Shoufeng Liu, Chunlin Gao, Xiaoguang Tong, Jialing Wu

**Affiliations:** ^1^Department of Neurorehabilitation and Neurology, Tianjin Huanhu Hospital, Tianjin Key Laboratory of Cerebrovascular and Neurodegenerative DiseasesTianjin, China; ^2^Department of Neurosurgery, Tianjin Huanhu Hospital, Tianjin Key Laboratory of Cerebrovascular and Neurodegenerative DiseasesTianjin, China

**Keywords:** high-sensitivity c-reactive protein, outcome, small-artery occlusion, ages, predictor

## Abstract

**Background:** High-sensitivity C-reactive protein (hs-CRP) is not only a marker of inflammation but also a prognostic factor for ischemic stroke. The objective of our study was to investigative the association between hs-CRP levels and outcomes of patients with small-artery occlusion (SAO).

**Methods:** We selected 718 participants diagnosed with SAO (according to Trial of Org 10172 in Acute Stroke Treatment classification) using the stroke registry of the Department of Neurorehabilitation of Tianjin HuanHu Hospital. Hs-CRP values at admission were classified into 3 categories: <0.91 mg/L, 0.91 to <2.77 mg/L, and ≥2.77 mg/L. Patients were divided into two subgroups based on age: the younger subgroup (<75years) and the elder subgroup (≥75 years). Clinical outcomes were evaluated with the modified Rankin scale (mRS) 3 months after the onset of stroke. We examined the relationship between hs-CRP levels at the time of admission and mRS scores using multivariate logistic regression analysis. We also assessed the association between hs-CRP levels and patient outcomes according to age.

**Results:** Among 718 patients with SAO (mean age, 61.7 ± 11.3 years), median hs-CRP was 1.54 mg/L. Although 628 patients had a favorable outcome, and 90 patients had a poor outcome at 3 months after SAO. Compared with the lowest levels of hs-CRP, those highest levels of hs-CRP (hs-CRP > 2.77 mg/L) were at increased risk of poor outcome (adjusted odds ratio, 1.917; 95% CI, 1.050–3.500; *P* = 0.034), and more than twice the risk of poor outcome among patients in the younger subgroup (adjusted odds ratio, 2.092; 95% CI, 1.079–4.058; *P* = 0.029). These associations persisted after adjustment for confounding risk factors. However, hs-CRP levels were not significantly associated with outcome among patients in the elder subgroup.

**Conclusions:** Elevated hs-CRP in patients with SAO is an independent predictor of poor prognosis; however, this association is only present in younger patients (<75 years).

## Introduction

Strok is an important health issue for individuals and society (Di Napoli et al., 2005). Ischemic stroke accounts for 70–80% of all stroke cases and is associated with high mortality and morbidity rates (Bonita et al., 2004). In terms of the Trial of Org 10172 in Acute Stroke Treatment (TOAST), small-artery occlusion (SAO) is a subtype of cerebral infarction. SAO, which is caused by occlusion of the deep branch arteries, accounts for 25% of ischemic stroke cases and occurs deep in the white matter, basal ganglia, or pons (Lavallée et al., 2013). Although the pathophysiology of cerebral small vessel disease is not clear, increased age, and hypertension and endothelial dysfunction are considered the main risk factors (van Dijk et al., 2005; Wada et al., 2008; Lavallée et al., 2013).

There is increasing evidence that elevated high-sensitivity C-reactive protein (hs-CRP) is a risk factor as well as a prognostic factor for ischemic stroke and coronary events (Rifai and Ridker, 2001; Di Napoli et al., 2005; Ishikawa et al., 2007; den Hertog et al., 2009; Song et al., 2009). Hs-CRP is a sensitive marker of inflammation and tissue injury in the arterial wall (Pearson et al., 2003; Pfützner and Forst, 2006). An hs-CRP assay quantifies small incremental changes in inflammation, which may be more clinically relevant compared with C-reactive protein for assessing the relationship between acute inflammation and predicting the degree of long-term disability (VanGilder et al., 2014). Although many studies have demonstrated that hs-CRP may affect ischemic stroke patients' outcomes, the association between hs-CRP and outcomes following SAO has not been established, especially among elderly patients diagnosed with SAO. Therefore, the aim of the present study was to investigate the association between hs-CRP levels and SAO outcomes according to patients' ages.

## Materials and methods

### Subjects

This study was approved by the ethics committee of Tianjin Huanhu Hospital and met the principles set forth in the 1964 Declaration of Helsinki and its later amendments or comparable ethical standards. An written informed consent was obtained from the participants and their families at the beginning of the study. All patients were screened according to a strict protocol consisting of complete medical history, full neurological examination, standardized blood tests, imaging examination, and a cardiac analysis that included standard 12-lead echocardiography. Detailed baseline data were collected and abstracted using electronic-based record forms during patients' hospitalization.

**Figure 1:**
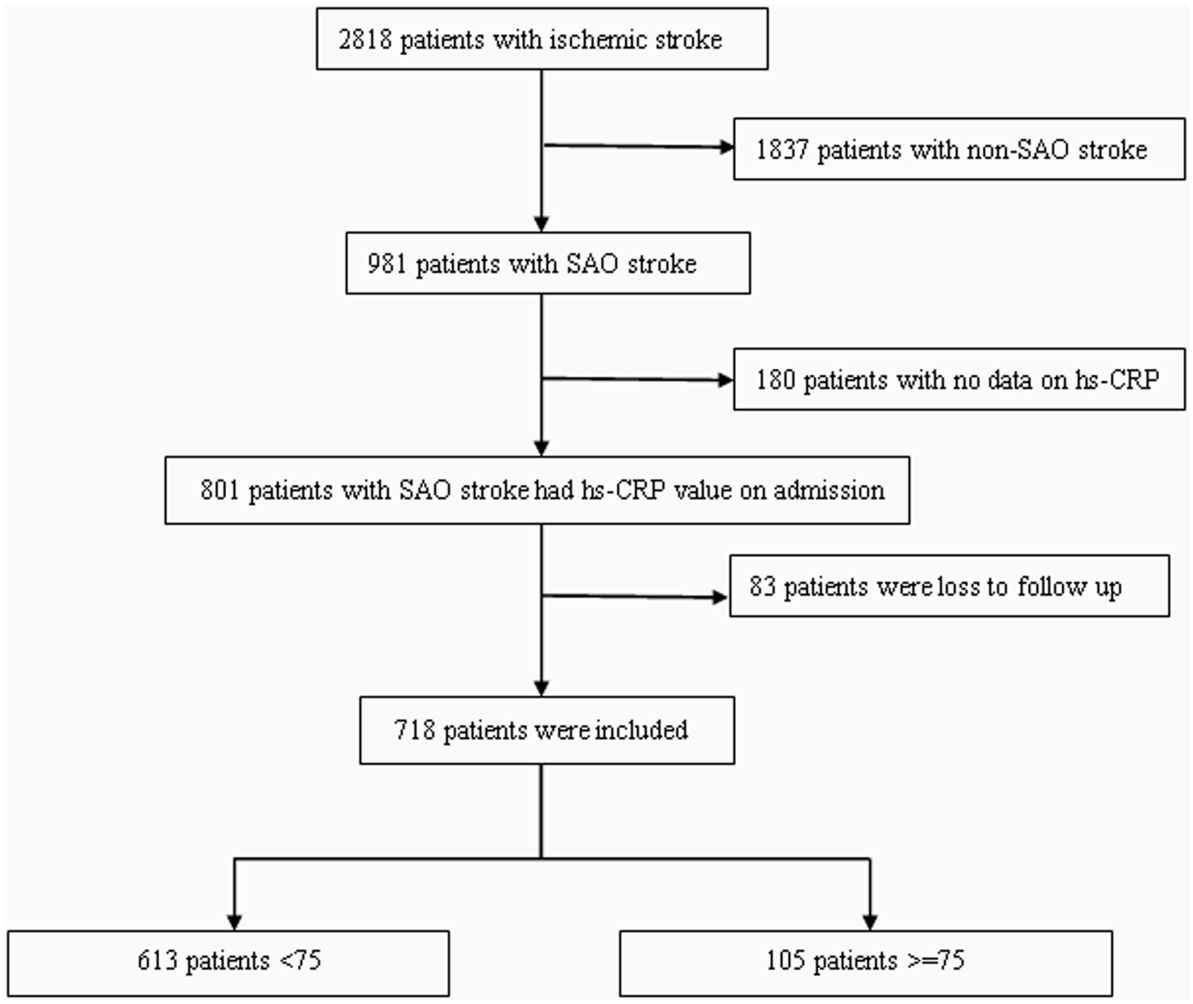
**Flow chart of patient selection**.

This study utilized a hospital-based registry of consecutive patients with stroke who visited the Department of Neurorehabilitation of Tianjin HuanHu Hospital between October 25, 2012 and June 30, 2015. There were 2818 cases of ischemic stroke diagnosed within 7 days of stroke onset; among these, 1837 were diagnosed with non-SAO, 180 patients had no hs-CRP data, and 83 patients were lost to follow-up. Finally, 718 patients with SAO met the inclusion criteria and were analyzed in this study (Figure 1). Finally, patients were classified into two subgroups according to age (younger subgroup, <75 years, *n* = 613; elder subgroup, ≥75 years, *n* = 105).

### Diagnostic criteria of SAO

The most widely applied classification is the TOAST subtype classification, which categorizes stroke into five etiological subtypes (Fromm et al., 2016), one of which is SAO. This category includes stroke cases that are often labeled as lacunar infarcts in other classification systems (Bamford et al., 1987). The diagnostic criteria of SAO are as follows: patients were required to have one of the traditional clinical lacunar syndromes with no evidence of cerebral cortical dysfunction; the infarction was required to have been located in the subcortex or brain stem and be <1.5 cm in diameter on computed tomography/magnetic resonance imaging; a history of diabetes mellitus or hypertension supported the clinical diagnosis; and patients were required to have no potential cardiac sources for embolism and stenosis no greater than 50% in an ipsilateral artery (Adams et al., 1993). Stroke cases were classified by two neurologists.

### Hs-CRP and clinical assessment

Blood samples were collected from the cubital vein of each patient before breakfast in the early morning within 24 h of admission (3.7 ± 2.1 days after symptom onset) (VanGilder et al., 2014). Hs-CRP assays were performed using immunoturbidimetric assay. Hs-CRP levels were classified into three groups: < 0.91 mg/L (*n* = 236), 0.91 to < 2.77 mg/L (*n* = 243), and ≥2.77 mg/L (*n* = 239). We conducted a laboratory calibration study to evaluate possible differences in the hs-CRP measurements between laboratories, specimen types, assay methods, instruments, and time of measurement, and found that the differences in hs-CRP were not large enough to warrant calibration.

Baseline information, including sex, age, and complete medical history, were recorded for each patient at admission; fasting glucose level, total cholesterol level, and other indicators were measured within 24 h of admission using a certified central laboratory. According to the Joint National Committee VI–VII, hypertension was defined as systolic blood pressure >140 mmHg and/or diastolic blood pressure >90 mmHg, based on the average of two blood pressure measurements, or a patient's self-reported history of hypertension or anti-hypertensive medication use, supported by documents (Munshi et al., 2008). Diabetes mellitus was diagnosed if the fasting plasma glucose level was >110 mg/dl or if the patient was taking anti-diabetic medications. Dyslipidemia was defined as either receiving treatment with cholesterol-reducing agents or a high-density lipoprotein cholesterol level ≤ 1.04 mmol/L, a triglyceride level ≥ 2.26 mmol/L, or a total cholesterol level ≥6.22 mmol/L. Patients who smoked tobacco products almost every day for >1 year were defined as current smokers. Patients who consumed alcohol at least one time per week for >1 year were defined as current drinkers. Body mass index was measured at the time of admission, and obesity was defined as a body mass index ≥30 kg/m^2^ (Wilterdink et al., 2001). An index of severity was obtained by all patients on the National Institutes of Health Stroke Scale (NIHSS) (Brott et al., 1989).

### Follow-up

Follow-up telephone interviews or in-person interviews were conducted with all the patients at 3 months after stroke onset. All follow-up results were recorded into the stroke database.

### Study outcome

Clinical outcomes were evaluated with the modified Rankin scale (mRS) 3 months after the onset of stroke. Patients were divided into two groups according to median mRS scores. Favorable outcome was defined as a score of 0–2, and poor outcome was defined as a score of 3–5. There were no records of patient deaths during the follow-up period.

### Statistical analysis

The distribution of hs-CRP levels and other risk factors were evaluated, for all patients and by patient groups. Other risk factors, such as age, sex, hypertension, diabetes, dyslipidemia, alcohol consumption, smoking status, obesity, and NIHSS score, were included in the univariate analysis as confounding factors. The relationship of hs-CRP levels with stroke outcomes was evaluated using multiple logistic regression analysis, which included confounding variables that were identified as significant (*P* < 0.05) in the univariate analysis.

Continuous variables are expressed as means ± standard deviations or tertiles. Differences between groups were analyzed for statistical significance using *t*-tests and one-way analysis of variance. Nominal variables were expressed as proportions. Differences in nominal variables between groups were assessed by using the chi-square test or Fisher's exact test. All tests were two sided and *P*-values < 0.05 were considered statistically significant. Statistical analyses were performed using the Statistical Package for Social Sciences (SPSS 17.0).

## Results

### Baseline demographics and clinical characteristics according to hs-CRP stratifications

Between October 25, 2012 and June 30, 2015, 718 patients (502 men [69.9%]; range, 30–83 years) diagnosed with SAO were identified. The baseline demographics and clinical characteristics of patients are presented in Table 1. NIHSS scores ranged from 1 to 14. The median (quartiles) hs-CRP values of all patients was 1545 mg/L (0.70, 3.71 mg/L). The median (quartiles) hs-CRP values of the three hs-CRP groups [<0.91 mg/L (*n* = 236), 0.91 to <2.77 mg/L (*n* = 243), and ≥2.77 mg/L (*n* = 239)] were 0.50 mg/L (0.32, 0.69 mg/L), 1.54 mg/L (1.22, 2.00 mg/L), and 5.24 mg/L (3.71, 10.00 mg/L), respectively. Hypertension, diabetes, and obesity were more prevalent in the group with the highest hs-CRP levels; however, the prevalence of alcohol drinkers was lowest in the group with the highest hs-CRP levels. Lower high-density lipoprotein cholesterol levels and higher HbA1c levels were associated with patients with the highest hs-CRP levels.

**Table 1:** **Baseline demographics and clinical characteristics according to hs-CRP groups**.

	**Groups of hs-CRP mg/L**		
	**<0.91 (*n* = 236)**	**0.91 to <2.77 (*n* = 243)**	**≥2.77 (*n* = 239)**
Age, years (median values)^*^	59.17 ± 10.75	61.37 ± 11.16	64.27 ± 11.44
Male sex, *n*(%)	175 (74.2)	163 (67.1)	164 (68.6)
**RISK FACTORS**			
Hypertension, *n*(%)^*^	163 (69.1)	185 (76.1)	198 (82.8)
Dyslipidemia, *n*(%)	62 (26.3)	72 (29.6)	61 (25.5)
Diabetes, *n*(%)^*^	83 (35.2)	119 (49.0)	125 (52.3)
Smokers, *n*(%)	108 (45.8)	83 (34.2)	92 (38.5)
Alcohol drinkers, *n*(%)^*^	57 (24.2)	40 (16.5)	38 (15.9)
Obesity, *n*(%)^*^	16 (6.8)	30 (12.3)	37 (15.5)
**LABORATORY FINDINGS**			
TC,mmol/L (median values)^*^	4.91 ± 0.95	4.82 ± 0.88	5.23 ± 2.89
TG,mmol/L (median values)	1.74 ± 1.12	1.66 ± 0.94	1.80 ± 1.14
HDL-C,mmol/L (median values)^*^	1.10 ± 0.28	1.05 ± 0.27	1.03 ± 0.24
LDL-C,mmol/L (median values)	2.94 ± 0.75	2.95 ± 0.69	3.08 ± 0.92
HbA1c,mmol/L (median values)^*^	6.42 ± 1.31	6.61 ± 1.24	6.82 ± 1.44
Hcy media, 25th percentile, 75th percentile)	11.80 (9.72, 15.45)	11.20 (9.38, 16.50)	12.00 (9.67, 17.10)
**NIHSS, n(%)**			
0~6	193 (81.8)	197 (81.1)	183 (76.6)
≥7	43 (18.2)	46 (18.9)	56 (23.4)

* *Indicates P < 0.05 when comparing between three groups. TC, total cholesterol; TG, triglyceride; HDL-C, high-density lipoprotein cholesterol; LDL-C, low-density lipoprotein cholesterol; HbA1c, hemoglobin A1c; hs-CRP, hypersensitive C-reactive protein; Hcy, homocysteine; NIHSS, National Institute of Health stroke scale*.

### Comparison of the risk factors between different groups classified by outcomes

Patients were divided into two groups according to mRS scores: the favorable outcome (score of 0–2) and poor outcome (score of 3–5) groups (Table 2). Risk factors, including age, sex, diabetes mellitus, smoking status, alcohol consumption, hs-CRP levels, and NIHSS scores, were significantly different between groups (*P* < 0.001, *P* < 0.028, *P* < 0.041, *P* < 0.008, *P* < 0.010, *P* < 0.003, and *P* < 0.001, respectively). During hospitalization, 22 patients developed infectious diseases (12 respiratory tract infections, 8 urinary tract infections, and two other infections), including 18 patients (2.9%) with favorable outcome and five patients (4.4%) with poor outcome. There was no statistical association in the distribution between the two parts (*P* = 0.627). Patients were also classified into two subgroups according to age [younger subgroup, <75 years (*n* = 613); elder subgroup, ≥75 years (*n* = 105)]. Sex, smoking status, alcohol consumption were significantly different between the younger subgroups with and without a favorable outcome (*P* < 0.045, *P* < 0.043, *P* < 0.034, respectively). Meanwhile, compared with unfavorable outcomes patients, patients with favorable outcomes were more likely have lower hs-CRP levels, and NIHSS scores in the younger subgroups (*P* < 0.024 and *P* < 0.001, respectively). However, hs-CRP levels between patients with and without a favorable outcome were not significantly different in the elder subgroups (*P* = 0.185).

**Table 2:** **Comparison of the risk factors between different groups classified by outcomes**.

	**Total (*****n*** **= 718)**			**≤75 (*****n*** **= 613)**			**≥75 (*****n*** **= 105)**		
	**mRS ≤ 2 (*n* = 628)**	**mRS ≥ 3 (*n* = 90)**	***p*-value**	**mRS ≤ 2 (*n* = 550)**	**mRS ≥ 3 (*n* = 63)**	***p*-value**	**mRS ≤ 2 (*n* = 78)**	**mRS ≥ 3 (*n* = 27)**	***p*-value**
Age, years (median values)	60.93 ± 11.01	66.38 ± 12.21	<0.001	58.39 ± 9.17	60.21 ± 8.78	0.134	78.87 ± 3.79	80.78 ± 4.27	0.032
Male sex, *n*(%)	448 (71.3)	54 (60.0)	0.028	406 (73.8)	69 (61.9)	0.045	42 (53.8)	15 (55.6)	0.878
Hypertension, *n*(%)	476 (75.8)	70 (77.8)	0.680	420 (76.4)	49 (77.8)	0.802	56 (71.8)	21 (77.8)	0.545
Dyslipidemia, *n*(%)	174 (27.7)	21 (23.3)	0.383	158 (28.7)	14 (22.2)	0.276	16 (20.5)	7 (25.9)	0.558
Diabetes, *n*(%)	277 (44.1)	50 (55.6)	0.041	245 (44.5)	34 (54.0)	0.155	32 (41.0)	16 (59.3)	0.101
Smokers, *n*(%)	259 (41.2)	24 (26.7)	0.008	248 (45.1)	20 (31.7)	0.043	11 (14.1)	4 (14.8)	0.927
Alcohol drinkers, *n*(%)	127 (20.2)	8 (8.9)	0.010	125 (22.7)	7 (11.1)	0.034	2 (2.6)	1 (3.7)	0.759
Obesity, *n*(%)	68 (10.8)	15 (16.7)	0.105	58 (10.5)	7 (11.1)	0.890	10 (12.8)	8 (29.6)	0.046
Hcy (media, 25th percentile, 75th percentile)	11.60 (9.63, 15.80)	12.20 (9.11, 17.50)	0.471	11.60 (9.53, 15.6)	11.70 (8.90, 20.03)	0.668	11.95 (10.20, 16.08)	13.00 (10.08, 16.50)	0.862
hs-CRP, *n*(%)			0.003			0.024			0.185
<0.91	216 (34.4)	20 (22.2)		199 (36.2)	17 (27.0)		17 (21.8)	3 (11.1)	
0.91 to <2.77	217 (34.6)	26 (28.9)		190 (34.5)	17 (27.0)		27 (34.6)	9 (33.3)	
≥2.77	195 (31.1)	44 (48.9)		161 (29.3)	29 (46.0)		34 (43.6)	15 (55.6)	
NIHSS, *n*(%)			<0.001			<0.001			0.036
0~6	527 (83.9)	46 (51.1)		472 (85.8)	33 (52.4)		55 (70.5)	13 (48.1)	
≥7	101 (16.1)	44 (48.9)		78 (14.2)	30 (47.6)		23 (29.5)	14 (51.9)	

*mRS, modified Rankin scale; hs-CRP, hypersensitive C-reactive protein; NIHSS, National Institute of Health stroke scale*.

### Multivariate logistical regression analyses of hs-CRP groups and outcome

Among all patients, compared to the lowest hs-CRP group, the highest hs-CRP group was independently associated with poor outcome at 3 months in the multivariate analysis, after adjusting for age, sex, diabetes mellitus, smoking status, alcohol consumption, and NIHSS score (adjusted odds ratio, 1.917; 95% CI, 1.050–3.500; *P* = 0.034; Table 3). However, the middle hs-CRP group (0.91 to < 2.77 mg/L) was not associated with poor outcome (*P* = 0.673). In the younger subgroup, compared with the lowest hs-CRP group, the highest hs-CRP group was also independently associated with poor outcome at 3 months in the multivariate analysis, after adjusting for sex, smoking status, alcohol consumption, and NIHSS score (adjusted odds ratio, 2.092; 95% CI, 1.079–4.058; *P* = 0.029); the middle hs-CRP group (0.91 to < 2.77 mg/L) was not associated with poor outcome (*P* = 0.961). However, in the elder subgroup, hs-CRP level was not associated with outcome after adjusting for risk factors (i.e., age, obesity, and NIHSS score) identified as significant in the univariate analysis (*P* = 0.341 and *P* = 0.263, respectively).

**Table 3:** **Multivariate logistical regression analyses of hs-CRP groups and the outcome variables**.

	**Total (*****n*** **= 718)**		**≤ 75 (*****n*** **= 614)**		**≥75 (*****n*** **= 105)**	
	**OR (95%CI)**	***p*-value**	**OR (95%CI)**	***p*-value**	**OR (95%CI)**	***p*-value**
Age	1.025 (1.002–1.048)	0.032			1.130 (1.009–1.265)	0.035
Male	1.175 (0.701–1.970)	0.541	1.269 (0.684–2.354)	0.449		
Diabetes	1.294 (0.803–2.085)	0.289				
Smokers	0.882 (0.487–1.598)	0.678	0.829 (0.431–1.595)	0.574		
Alcohol drinkers	0.638 (0.273–1.490)	0.299	0.599 (0.244–1.472)	0.264		
Obesity					2.902 (0.945–8.919)	0.063
**hs-CRP**						
<0.91	index		index		index	
0.91 to <2.77	1.148 (0.605–2.178)	0.673	2.334 (0.474–2.037)	0.961	2.105 (0.455–9.734)	0.341
≥2.77	1.917 (1.050–3.500)	0.034	2.092 (1.079–4.058)	0.029	2.283 (0.537–9.696)	0.263
NIHSS	4.114 (2.531–6.687)	<0.001	5.211 (2.972–9.138)	<0.001	2.206 (0.848–5.738)	0.105

*mRS, modified Rankin scale; hs-CRP, hypersensitive C-reactive protein; NIHSS, National Institute of Health stroke scale*.

## Discussion

We evaluated patient information using a hospital-based stroke registry from Tianjin, northern China. To the best of our knowledge, this is the first study that has aimed to assess the association between plasma hs-CRP levels and 3 month outcomes among SAO patients in northern China. Compared with those patients from southern China, our patients have lower age (61 vs. 69 years), lower plasma hs-CRP levels (1.55 vs. 3.35 mg/L), more Diabetes (45.5 vs. 19.14%) (Lv et al., 2016).

In our study, elevated hs-CRP levels in patients with SAO was an independent predictor of poor prognosis 3 months after SAO; however, this association was only present in the younger subgroup. In line with our findings, the Circulatory Risk in Communities Study reported a positive association between hs-CRP levels and lacunar stroke in Japanese men and women (Chei et al., 2011). Rajeshwar et al. (2012) and Ladenvall et al. (2006) found no significant association between CRP levels and the prognosis of patients with lacunar infarcts after 3 months. These contradictory findings may result from differences in sample sizes, patient race, patient age, methods used to evaluate disease, and analytic strategies. Moreover, advanced age, hypertension, diabetes, alcohol consumption, obesity, total cholesterol levels, lower high-density lipoprotein cholesterol levels, and higher HbA1c levels were associated with elevated hs-CRP levels (Latha and Chandrakala Shenoy, 2008; Wada et al., 2008; Jiang et al., 2009; Chaudhuri et al., 2013). This is in accordance with the findings of the current study.

In this study, elevated hs-CRP levels were associated with prognosis in younger patients but not in older patients. Although the pathophysiological link between cerebral small-vessel disease and hs-CRP is not clear, different mechanisms may be involved. Hs-CRP is a marker of inflammation, and the inflammatory response plays an important role in patients with SAO; it is associated with plaque instability, stenosis, or occlusion of the small vessels (Zeng et al., 2013). On the other hand, CRP levels may be a marker for arteriolosclerosis or small-vessel disease, as they are for atherosclerosis. Arteriolosclerosis may result in lacunar infarcts through vessel occlusion, disturbed cerebral autoregulation, or increased vascular permeability (Pantoni and Garcia, 1997). More than one mechanism or a combination of mechanisms could explain the association between high CRP levels and SAO. Many studies have demonstrated that high levels of inflammatory mediators, such as CRP, may be used as prognostic indicators after lacunar stroke (Elkind et al., 2014).

In this study, there was no significant association between hs-CRP and outcome among elderly patients. This may be because aging is associated with weakened innate and adaptive immunity, both quantitatively and qualitatively (Gao et al., 2014). Although Matsuo et al found that an elevated plasma hs-CRP levels could predict poor functional outcome at 3 months, however, odds ratios for poor functional outcome increased more markedly as an elevation of plasma hs-CRP levels in younger patients (aged < 70 years) than in those aged over 70 years (Matsuo et al., 2016). Matsuo et al. study recruited not only SAO patients but also other TOAST subtype ischemic stroke patients, but in line with present study that inflammation seems have more marked effect on stroke patients' outcome in younger patients. Thus, the inflammation reaction may have been weak among the elderly patients included in the present study; similarly, the impact of inflammation in the pathogenesis of small vessel disease-related brain lesions seems to be weak among the Japanese elderly (Wada et al., 2008). Second, the elderly patients are more likely to be taking statins, aspirin, or NSAIDs on a regular basis, and this would explain lower inflammatory activity in these patients, they are potential candidates to attenuate progression of lesions related to small vessel disease and their consequences (Ridker et al., 2001). Previous studies by van Dijk et al. (2005) and Wada et al. (2008) found no significant relationship between CRP levels and small vessel disease severity in the elderly. The two previous studies assessed the relationship between CRP levels and small vessel disease severity in the elderly, but we assessed the relationship between hs-CRP levels and prognosis of SAO in the elderly. Furthermore, Wada and colleagues recruited patients with not only lacunar infarcts but also white matter hyperintensity (Wada et al., 2008). These lesions are commonly observed on magnetic resonance imaging scans in elderly people and are associated with an increased risk of stroke (Vermeer et al., 2003). Age and ethnicities of patients included in the studies differed, and hs-CRP levels may vary among races and ethnic groups (Albert et al., 2004; Khera et al., 2005). Albert et al. previously reported that median hs-CRP values among Asian women were significantly lower than those among other races and ethnic groups in the United States (Albert et al., 2004).

The present study had certain limitations. First, as all the subjects were recruited from a group of individuals who voluntarily participated in the system, the sample population does not represent the general population of China. Second, 718 patients with SAO were involved in this study and no death record had been found during the follow-up. In fact, it is impossible for all the patients to achieve a mortality rate of zero, although SAO has the lowest mortality rate among stroke subtypes and we chose a short follow-up period. Therefore, there may be a selection bias which is inevitable. Third, the timing of sampling in relation to stroke onset may have impacted our findings; further prospective studies of the optimal timing of CRP measurement for use as a prognostic marker after ischemic stroke are warranted. Although extensive prospective studies are necessary to establish the link between hs-CRP and small vessel disease, our study clearly demonstrated the association between hs-CRP levels and the outcomes of SAO patients according to age.

## Conclusion

In the present study, we can conclude that elevated hs-CRP levels in patients with SAO is an independent predictor of poor prognosis 3 months after SAO; this association was present in younger, but not in older patients. The underlying mechanisms have yet to be determined and our findings await confirmation in other populations.

## Author contributions

JW contributed to the conception and design of the work; RQ, YG, DH, YW, CY, WW, SL, and CG contributed the data acquisition; XT and JW contributed the analysis and interpretation of data for the work; CY contributed drafting the work, JW contributed revising the work for important intellectual content.

### Conflict of interest statement

The authors declare that the research was conducted in the absence of any commercial or financial relationships that could be construed as a potential conflict of interest.
